# Turing Universality of Weighted Spiking Neural P Systems with Anti-spikes

**DOI:** 10.1155/2020/8892240

**Published:** 2020-09-17

**Authors:** Qianqian Ren, Xiyu Liu, Minghe Sun

**Affiliations:** ^1^Academy of Management Science, Business School, Shandong Normal University, Jinan, China; ^2^College of Business, The University of Texas at San Antonio, San Antonio, TX, USA

## Abstract

Weighted spiking neural P systems with anti-spikes (AWSN P systems) are proposed by adding anti-spikes to spiking neural P systems with weighted synapses. Anti-spikes behave like spikes of inhibition of communication between neurons. Both spikes and anti-spikes are used in the rule expressions. An illustrative example is given to show the working process of the proposed AWSN P systems. The Turing universality of the proposed P systems as number generating and accepting devices is proved. Finally, a universal AWSN P system having 34 neurons is proved to work as a function computing device by using standard rules, and one having 30 neurons is proved to work as a number generator.

## 1. Introduction

Membrane computing, introduced by Păun [1], is a branch of nature-inspired computing. It provides a rich computational framework for biomolecular computing. Models of membrane computing are inspired by the structures and functions of living cells. The obtained models are distributed and parallel computing devices, usually called P systems [2]. There are three main classes of P systems: cell-like P systems, tissue-like P systems [3], and neural-like P systems [4]. Neural-like P systems, inspired by the ways of information storage and processing in human brain nervous systems, are systems that combine neurons and membrane computing, among which the most widely known are spiking neural P systems (SN P systems) [5]. A SN P system consists of a group of neurons located at the nodes of a directed graph, and neurons send spikes to adjacent neurons through synapses, i.e., links in the graph. There is only one type of objects, i.e., spikes, in the neurons.

With different biological features and mathematical motivations, many variants of SN P systems have emerged. Some of them made changes on synapses between neurons, such as SN P systems with rules on synapses [6], SN P systems with multiple channels [7], and SN P systems with thresholds [8], while others made changes on the communication rules, such as SN P systems with communication on request [9], SN P systems with polarizations [10], and SN P systems with inhibitory rules [11]. Various new variants of SN P systems are provided in [12, 13]. Recently, some new variants of neural-like P systems have been proposed, which are inspired by SN P systems, such as those reported in [14]. In addition, many publications appeared in the literature on the computational power of SN P systems as function computing devices and the number generating/accepting devices. Pǎun [18] proved small universality of SN P systems. Pan [19] proved the small universality of SN P systems with communication on request by using 14 neurons, and more details are available in [20, 21].

Since the SNP system was proposed, many scholars have explored its applications. At present, there are many applications of SN P systems, such as skeletonizing image processing [22, 23], optimization problems [24], fault diagnosis [25–27], and working models [28].

Inspired by the spikes of inhibition of communication between neurons, a new type of SN P systems is proposed by adding anti-spikes to SN P systems, which is called spiking neural P systems with anti-spikes (ASN P systems) [29]. In ASN P systems, each neuron contains multiple copies of symbolic object *a* or $\bar{a}$ and processes information by spiking rules and forgetting rules. The annihilating rule $a\bar{a}⟶\lambda$ exists in each neuron and is the first to apply, meaning *a* and $\bar{a}$ cannot coexist in any neuron. Many researchers have proposed different ASN P systems, such as ASN P systems with multiple channels [30], ASN P systems with rules on synapses [31], and asynchronous ASN P systems [32]. The computational power of ASN P systems as number generating and accepting devices, as well as function computing devices, also can be proved [33].

In [34], SN P systems with weighted synapses were proposed. The weights represent the numbers of synapses between connected neurons. Based on the above, a new variant of SN P systems, called the weighted spiking neural P systems with anti-spikes (AWSN P systems),is proposed in this work. In these systems, neurons receive spikes or anti-spikes from their connected neurons and the numbers of spikes or anti-spikes they receive are determined by the weights of the synapses. Only one type of objects, i.e., spikes or antispikes, exists in each neuron with standard rules in SN P systems. These systems use spiking rules with the form of (*E*/*a*^*c*^)⟶*a*^*p*^; *d* (called standard rules if *p* = 1 and extended rules otherwise), where *E* is a regular expression over spikes *a* and *c*, and*p* and *d* are all positive integers. The meaning of the spiking rules is that *c* spikes are consumed and *p* spikes are generated after *d* time periods. SN P systems also have forgetting rules of the form *a*^*s*^⟶*λ*, where *s* is a positive integer. The meaning of the forgetting rules is that *s* spikes are dissolved or removed from a neuron.

The rest of this article is organized as follows. In Section 2, the basic knowledge of a register machine is given. The definition of AWSN P systems is given, and an example is presented to show their working process in Section 3. By simulating register machines, the computational power of AWSN P systems is proved as natural number generating devices and accepting devices in Section 4. In Section 5, the universality of these systems as function computing devices and number generating devices is obtained by using 34 neurons and 30 neurons, respectively. Remarks and future research directions are given in Section 6.

## 2. Prerequisites

The universality of systems is proved by simulating a register machine *M*. A register machine is structured as *M*=(*m*, *H*, *l*_0_, *l*_*h*_, *I*), where *m* is the number of registers, *H* is the set of instruction labels, *l*_0_ and *l*_*h*_ are the starting and ending labels, and *I* is the set of instructions shown below:

1. *l*_*i*_ : (ADD(*r*), *l*_*j*_, *l*_*k*_) (add 1 to register *r* and then go to instruction labels *l*_*j*_ or *l*_*k*_ with nondeterministic choice)
2. *l*_*i*_ : (SUB(*r*), *l*_*j*_, *l*_*k*_) (if register *r* is not empty, then subtract 1 from it and go to *l*_*j*_; otherwise, go to *l*_*k*_)
3. *l*_*h*_ : HALT (the ending instruction)

A register machine has two modes: a generating mode and an accepting mode. A register machine *M* generates a set of numbers indefinitely, denoted by *N*_gen_(*M*) , and works in the following way in the generating mode. When all the registers start empty, *M* starts the computational process from the instruction label *l*_0_. When *M* reaches *l*_*h*_, the computation ends with the results stored in register 1. If the computation does not stop, the numbers will not be generated. A set of numbers can also be accepted by a register machine, denoted as *N*_acc_(*M*), in the accepting mode. Only the input neuron is nonempty at the beginning. It then works in a way similar to that in the generating mode. As register machines are universal in the accepting mode, the add instructions can be written as *l*_*i*_ : (ADD(*r*), *l*_*j*_). Register machines can compute any set of Turing computable numbers represented by NRE (see, e.g., [6]).

**Figure 1:**
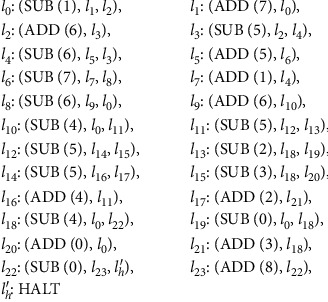
The universal register machine *M*_*u*_′.

Generally, a universal register machine is used to compute Turing computable functions for the purpose of analyzing the computing power of system. A universal register machine *M*_*u*_ is proposed by Minsky [35]. If *φ*_*x*_(*y*) = *M*_*u*_(*g*(*x*), *y*) satisfies that *x* and *y* are natural numbers and *g* is a recursive function, then *M*_*u*_ is universal, denoted by *M*_*u*_ = (8, *H*, *l*_0_, *l*_*h*_, *I*), including 8 registers and 23 instructions. Compared with register machine *M*_*u*_′ as shown in Figure 1, register machine *M*_*u*_ does not have instructions *l*_22_ and *l*_23_, and the final result is placed in register 0. Since the result is stored in register 0, it cannot contain any SUB instruction. Hence, register 8 is added and used to store the result without any SUB instruction. In general, in order to analyze the universality of the system, i.e., to verify that the system is equivalent to a Turing machine, a universal register machine *M*_*u*_′as shown in Figure 1 is simulated by a system, denoted by *M*_*u*_′ = (9, *H*, *l*_0_, *l*_*h*_, *I*), consisting of 9 registers and 25 instructions.

## 3. Weighted Spiking Neural P Systems with Anti-spikes

### 3.1. Definition

The proposed AWSN P system is described as follows:

(1) $$\begin{array}{c} \prod =\left(O,\sigma_{1},\sigma_{2},\ldots ,\sigma_{m},\text{syn},\text{in},\text{out}\right), \end{array}$$

where

(1) $O=\left\{a,\bar{a}\right\}$ is the set of alphabets, where the symbol *a* is a spike, and $\bar{a}$ is an anti-spike.
(2) *σ*_1_, *σ*_2_,…, *σ*_*m*_ are neurons, in the form of *σ*_*i*_ = (*n*_*i*_, *R*_*i*_) for 1 ≤ *i* ≤ *m*, where *n*_*i*_ ≥ 0 is the initial number of spikes stored in *σ*_*i*_, and *R*_*i*_ is the set of rules used in *σ*_*i*_ in the following form:
  - Spiking rules, (*E*/*b*^*c*^)⟶*b*^′*p*^; *d*, where *E* is a regular expression over *a* or $\bar{a}$, $b,b^{\prime }\in \left\{a,\bar{a}\right\}$, *c* ≥ *p* ≥ 1, and *d* ≥ 0 are the time unit
  - Forgetting rules, *b*^*s*^⟶*λ*, where $b\in \left\{a,\bar{a}\right\}$ and*s* ≥ 1
(3) syn ∈ {1,…, *h*} × {1,…, *h*} × *W* represents the synapses, where *W* = {1,…, *n*} is the set of weights. For any(*i*, *j*, *n*) ∈ syn, 1 ≤ *i*,  *j* ≤ *h*, *i* ≠ *j*, and *n* ∈ *W*.
(4) in and out are the input neuron and output neuron.

In the AWSN P system, each neuron has one or more spiking rules and some of them also have forgetting rules, and either spikes or anti-spikes exist in each neuron. If there are *k* spikes or anti-spikes in neuron *σ*_*i*_, *b*^*k*^ ∈ *L*(*E*) and *k* ≥ *c*, the spiking rule (*E*/*b*^*c*^)⟶*b*^′*p*^; *d* can be stimulated. If *k* = *c*, then the spiking rule is called pure, and the rule can be written as*b*^*c*^⟶*b*′; *d*. The spiking rule can be interpreted as follows. If *c* spikes or anti-spikes are removed from neuron *σ*_*i*_ and the neuron fires, *p* spikes will be generated after *d* time periods (as usual in membrane computing, all neurons in a system Π work in parallel with an assumed global clock) and *p* × *n* spikes will be sent to neuron *σ*_*j*_(*i* ≠ *j*), where *n* ∈ *W*. If the spiking rule of neuron *σ*_*i*_ is used in time *d* for all *d* ≥ 1, the neuron will be closed before time *t* + *d* and will not receive any spikes or anti-spikes, and then the neuron will open at time *t* + *d*. If *t* = 0, spikes will be emitted immediately, which means the neuron receives spikes or anti-spikes from the upper neuron without delay.

If the forgetting rules *b*^*s*^⟶*λ* in the neurons are used, then the *s* spikes or anti-spikes are removed from the neurons. Spiking rules and forgetting rules must be applied if the conditions are met, but the choice of rules is nondeterministic if the conditions of multiple rules are met in a neuron. However, the annihilating rule $a\bar{a}⟶\lambda$ must be applied first in each neuron.

Through these rules, transitions between configurations can occur. Any sequence of transitions starting from the initial configuration is called a computation. A computation will stop when it reaches a configuration where all neurons are open and no rules can be used. To compute the function *f* : *N*^*K*^⟶*N*, *k* natural numbers *n*_1_, *n*_2_ ⋯ , *n*_*k*_ are introduced into the system by reading a binary sequence *z*=10^*n*_1_^, 10^*n*_2_^1,…, 10^*n*_*k*_^1 from the environment. That is to say, the input neuron of Π receives a spike in a step if it corresponds to 1 in *z*, but it receives nothing if it corresponds to 0. The input neuron received exactly *k*+1 spikes and will not receive any more spikes after receiving the last spike. The result of the computation is encoded in the distance between two spikes, which means that the computation halts with exactly two spikes as outputs immediately after outputting the second spike. Hence, it generates a spike string of the form 0^*b*^10^*r*−1^1, for *b* ≥ 0 and *r*=*f*(*n*_1_,…, *n*_*k*_). The computation outputs no spike for a nonspecified number of steps from the beginning of the computation until outputting the first spike.

Let *N*_gen_(∏) and *N*_acc_(∏) be the sets of numbers generated and accepted by Π, respectively. Let *N*_*α*_ASN*P*_*m*_^*n*^, with *α* ∈ {gen, acc}, denote the family of sets of numbers generated or accepted by an AWSN P system with *m* neurons and a maximum of *n* rules in a neuron.

### 3.2. An Illustrative Example

**Figure 2:**
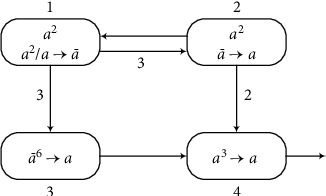
An example of the AWSN P system.

An example as graphically shown in Figure 2 is given to explain the working process of the AWSN P system. The results of each step are shown in Table 1. A positive number in the table represents the number of spikes in the neuron, and a negative number represents the number of anti-spikes. For example, 2 means there are two spikes, and −2 means there are two anti-spikes.

The system has four neurons as shown in Figure 2. Assume that each of neurons *σ*_1_ and *σ*_2_ has two spikes, and neurons *σ*_3_ and *σ*_4_ are empty with no spikes. Suppose that the rule $\left(a^{2}|/|a\right)⟶\bar{a}$ in neuron *σ*_1_ can be used at time *t*, generating one anti-spike and sending three anti-spikes to neurons *σ*_2_ and *σ*_3_ because the weight of synapses between these neurons is 3. Two anti-spikes together with two spikes disappear immediately because the annihilating rule is applied first, and there is one anti-spike left in neuron *σ*_2_. The rule in *σ*_2_ generates two spikes to be sent to neuron *σ*_4_ and one spike to be sent to neuron *σ*_1_. So the rule in *σ*_1_ can be applied again. Neuron *σ*_3_ receives six anti-spikes from *σ*_1_ by using the rule of neuron *σ*_1_ twice, so that the rule in *σ*_3_ fires. Neuron *σ*_4_ gets three spikes (two from neuron *σ*_2_) and sends one spike to the environment.

## 4. Computational Models

### 4.1. Generating Mode

**Theorem 1 .**

*N*_gen_ASN*P*_*∗*_^2^=NRE.

**Proof**

A register machine *M*=(*m*, *H*, *l*_0_, *l*_*h*_, *I*) is considered. *M* is simulated by an AWSN P system, including three modules, i.e., modules ADD, SUB, and OUTPUT.

In the simulation process, a register *r* of *M* corresponds to neuron*σ*_*r*_ , and the number *n* contained in register *r* is the number of spikes contained in neuron *σ*_*r*_. An instruction *l* in *H* corresponds to neuron *σ*_*l*_. Furthermore, the modules require some other neurons in addition to *σ*_*r*_ and *σ*_*l*_. The simulation of the ADD and SUB instructions begins at neuron *σ*_*l*_*i*__. Modules ADD and SUB are simulated by sending spikes to *σ*_*l*_*j*__ and *σ*_*l*_*k*__ as rules in neuron *σ*_*r*_ fire. Neuron *σ*_*r*_ sends a spike to either *σ*_*l*_*j*__ or *σ*_*l*_*k*__, but the choice is nondeterministic. When a spike arrives at neuron *σ*_*l*_*h*__, the computation in *M* stops, and the module OUTPUT begins to send the result stored in register 1 to the environment. At the beginning of the simulation, neuron *σ*_*l*_0__ has one spike but other neurons do not have any spikes.

**Figure 3:**
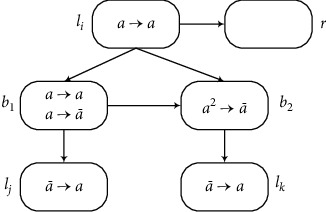
Module ADD: stimulating the ADD instruction *l*_*i*_ : (ADD(*r*), *l*_*j*_, *l*_*k*_).

**Figure 4:**
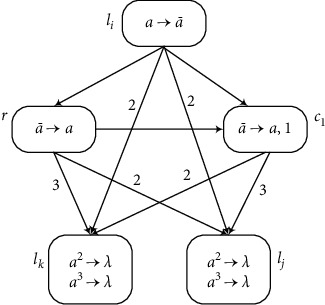
Module SUB: simulating the SUB instruction *l*_*i*_ : (SUB(*r*), *l*_*j*_, *l*_*k*_).

- Module ADD (Shown in Figure 3) Assume that an ADD instruction *l*_*i*_ : (ADD(*r*), *l*_*j*_, *l*_*k*_) has to be simulated at time *t*, one spike is in neuron *σ*_*l*_*i*__, and the rule *a*⟶*a* can be used. Neuron *σ*_*l*_*i*__ sends one spike *a* to neurons *σ*_*r*_, *σ*_*b*_1__, and *σ*_*b*_2__, respectively. The rules $a⟶\bar{a}$ and *a*⟶*a* in neuron *σ*_*b*_1__ are chosen in a nondeterministic way for use at time *t* + 1. In this way, there are two cases to consider depending on the choice of the rules in *σ*_*b*_1__. If *a*⟶*a* is chosen, neuron *σ*_*b*_2__ sends a spike to neuron *σ*_*l*_*k*__. Thus, *σ*_*l*_*k*__ will generate one spike by using its rule. If $a⟶\bar{a}$ is chosen, neuron *σ*_*b*_1__ sends an anti-spike to neurons *σ*_*l*_*i*__ and *σ*_*b*_2__, respectively. Thus *σ*_*l*_*j*__ will fire and generate one spike by using its rule. The rule in neuron *σ*_*b*_2__ cannot be used because of the annihilating rule, so that *σ*_*l*_*k*__ is empty. After one spike is added to *σ*_*r*_, the register *r* adds 1 and the instruction *l*_*j*_ or *l*_*k*_ is activated. Therefore, the ADD instruction can be simulated correctly by the module ADD.

(b) Module SUB (Shown in Figure 4) Suppose that neuron *σ*_*l*_*i*__ has one spike. After the rule $a⟶\bar{a}$ is enabled at time *t*, each of the neurons *σ*_*l*_*j*__ and *σ*_*l*_*k*__ receives two anti-spikes $\bar{a}$, and *σ*_*r*_ receives one anti-spike. The rest of the computation can be divided into two cases according to the number of spikes contained in *σ*_*r*_.

1. *Neuronσ*_*r*_ *has at least one spike*. Neuron *σ*_*r*_ receives one anti-spike from neuron *σ*_*l*_*j*__, but anti-spike will disappear immediately by annihilating one spike in *σ*_*r*_. Therefore, the rule $\bar{a}⟶a$ in neuron *σ*_*r*_ is not used at time *t* + 1. At the same time, neuron *σ*_*c*_1__ opens to get one anti-spike from *σ*_*l*_*i*__, and then the rule in *σ*_*c*_1__ fires and generates one spike but sends three spikes to neurons *σ*_*l*_*j*__ and two spikes to *σ*_*l*_*k*__. The two spikes are annihilated with two anti-spikes from *σ*_*l*_*i*__ and one spike is left in neuron *σ*_*l*_*j*__. Simultaneously, the same happens in neuron *σ*_*l*_*k*__, i.e., the two spikes are annihilated immediately and there is no spike left in *σ*_*l*_*k*__.

(2) *Neuronσ*_*r*_* has no spike*. Neuron *σ*_*r*_ gets one anti-spike from *σ*_*l*_*i*__ and its rule can be applied at time *t* + 1. Simultaneously, neuron *σ*_*c*_1__ gets one anti-spike from *σ*_*l*_*i*__. Hence, one spike from *σ*_*r*_ is annihilated in the next time. The rule in *σ*_*r*_ cannot be used because *σ*_*r*_ does not have any anti-spikes. At the same time, neuron *σ*_*l*_*j*__ receives five spikes, among which two spikes are used to annihilate the two anti-spikes received from neuron *σ*_*l*_*i*__; thus the rule *a*^2^⟶*λ* in *σ*_*l*_*j*__ can be applied. Neuron *σ*_*l*_*k*__ receives one spike that annihilates one anti-spike$\bar{a}$ received from neuron *σ*_*l*_*i*__, and then the rule $\bar{a}⟶a$ in *σ*_*l*_*k*__ is enabled to generate one spike *a*.

Therefore, the SUB instruction can be simulated correctly by module SUB.

**Figure 5:**
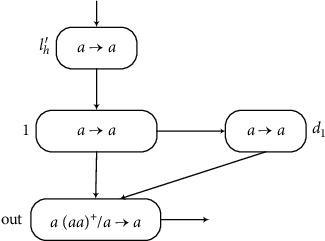
Module OUTPUT.

(c) Module OUTPUT (Shown in Figure 5) Assume that *σ*_*l*_*h*__ of system ∏ accumulated one spike at time *t*, and neuron *σ*_1_ has *n* spikes for the number *n* being stored in register 1 of *M*. When the rule in *σ*_*l*_*h*_′_ is fired at time *t*, neuron *σ*_*l*_*h*_′_ sends one spike to *σ*_1_. At this moment, *σ*_1_ has an odd number of spikes and its rule fires. At time *t* + 1, *σ*_1_ sends one spike to *σ*_*out*_ and *σ*_*b*_1__, respectively. Thus, neuron *σ*_out_ has one spike, which is an odd number. At time *t* + 2, neuron *σ*_out_ fires, sending one spike to the environment. At the same time, the rules in *σ*_1_ and *σ*_*b*_1__ are used, and both send one *a* to *σ*_out_. After *n* − 1 steps, until neuron *σ*_1_ has no spike, the number of spikes in *σ*_out_ is even. At the same time, the use of the rule in *σ*_1_ is stopped, and neuron *σ*_*b*_1__ has one spike. Neuron *σ*_out_ will receive one spike at time *t* + *n* + 2, and then the number of spikes is odd. Neuron *σ*_out_ fires a second time. Therefore, the number computed by the AWSN P system is the difference between the first two steps when the neuron *σ*_out_ fires; that is, (*t* + *n* + 2) − (*t* + 2) = *n*. The module OUTPUT can be simulated correctly.

### 4.2. Accepting Mode

**Theorem 2 .**

*N*_acc_ASN*P*_*∗*_^2^=NRE.

**Proof**

The proof of this theorem is similar to that of Theorem 1. A register machine *M*=(*m*, *H*, *l*_0_, *l*_*h*_, *I*), consisting of three modules, ADD, SUB, and INPUT, is considered. Module SUB is shown in Figure 4.

**Figure 6:**
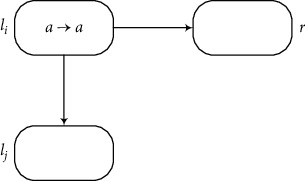
Module ADD: simulating the ADD instruction *l*_*i*_ : (ADD(*r*), *l*_*j*_).

**Figure 7:**
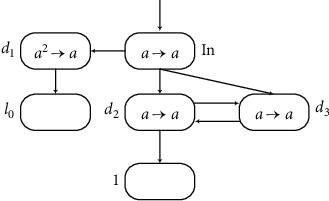
Module INPUT.

1. Module ADD (Shown in Figure 6) Assume that an ADD instruction *l*_*i*_ : (ADD(*r*), *l*_*j*_) has to be simulated at time *t*. Suppose that one spike is in neuron *σ*_*l*_*i*__; then the rule *a*⟶*a* can be used. Thus, neuron *σ*_*l*_*i*__ sends one spike to neurons *σ*_*r*_ and *σ*_*l*_*j*__. In this way, the number of spikes in *σ*_*r*_ increases by 1 and the instruction *l*_*j*_ is activated. Hence, the ADD instruction can be simulated correctly by this module.

(2) Module INPUT (Shown in Figure 7) Module INPUT shown in Figure 7 works as follows. The function of module INPUT is to read the spike train 10^*n*−1^1 and compute the number *n* in the time between receiving two spikes. When neuron *σ*_*in*_ receives the first spike at time*t* and then neurons *σ*_*d*_1__,*σ*_*d*_2__, and *σ*_*d*_3__ receive one spike each, the rule in *σ*_*d*_2__and *σ*_*d*_3__ can be applied at time*t* + 1. At time*t* + 2, neuron *σ*_1_ gets one spike, and, at the same time, neuron *σ*_*d*_3__ gets one spike from *σ*_*d*_2__ and neuron *σ*_*d*_2__ receives one *a* from *σ*_*d*_3__. Therefore, in the next *n* − 1time periods, the rules in neurons *σ*_*d*_2__ and *σ*_*d*_3__ can continued to be used. During this period, *σ*_1_ gets *n* − 1 spikes. When neuron *σ*_*in*_ receives the second spike at step *t* + *n*, each of neurons *σ*_*d*_2__ and *σ*_*d*_3__ receives one spike at step *t* + *n* + 1 and they both have two spikes. In this way, neurons *σ*_*d*_2__ and *σ*_*d*_3__ cannot fire to send any spikes to neuron *σ*_1_. In the whole process, neuron *σ*_1_ receives (*n* − 1) + 1 = *n* spikes, i.e., the number *n*is stored in register 1.

From the descriptions above about the three modules, it is clear that the register machine *M* can correctly simulate the system. The proof is complete.

## 5. A Small Universal AWSN P System

### 5.1. The Universality as Function Computing Devices

**Theorem 3 .**

There is a universal AWSN P system having 34 neurons which can be used to perform function computing.

**Proof**

**Figure 8:**
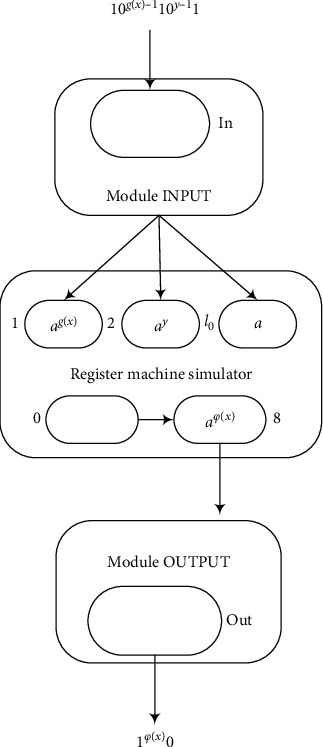
General framework of the universal AWSN P system.

**Figure 9:**
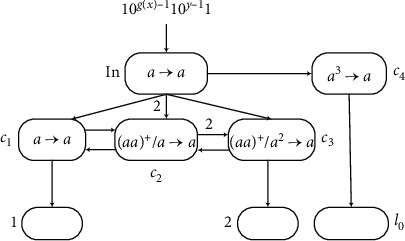
Module INPUT.

A general framework of a system ∏_*u*_′ used to simulate a universal register machine *M*_*u*_′ is shown in Figure 8, which is a universal AWSN P system. Π′ consists of 8 modules: ADD, SUB, ADD-ADD, SUB-ADD-1, SUB-ADD-2, SUB-SUB, INPUT, and OUTPUT. The modules SUB, OUTPUT, and ADD are the same as those in Figures 4–6, respectively. The module INPUT is shown in Figure 9.

Module INPUT works as follows: when neuron *σ*_in_ gets a spike from the environment, the rule *a*⟶*a* fires and one spike is sent to neurons *σ*_*c*_1__, *σ*_*c*_3__, and *σ*_*c*_4__, and two spikes are sent to neuron *σ*_*c*_2__. Then, the rule in neuron *σ*_*c*_1__ sends one spike to both *σ*_*c*_2__ and *σ*_1_. At the same time, neuron *σ*_*c*_2__ fires and then sends one spike to *σ*_*c*_1__ and two spikes to *σ*_*c*_3__. Up to this point, three spikes were sent to neuron *σ*_*c*_3__. Therefore, before neuron *σ*_in_ receives more spikes from the environment, neurons *σ*_*c*_1__ and *σ*_*c*_2__ have received one spike from each other in each time period and neuron *σ*_1_ has received *g*(*x*) spikes.

When *σ*_in_ receives the second spike, each of the neurons *σ*_*c*_1__, *σ*_*c*_3__, and *σ*_*c*_4__ can get one spike and *σ*_*c*_2__ gets two spikes. Neuron *σ*_*c*_2__ has four spikes at this moment, and its rule can be used to send two spikes to neuron *σ*_*c*_3__. Neuron *σ*_*c*_3__ then has six spikes, so that the rule in *σ*_*c*_3__ is used to produce one spike and send it to *σ*_*c*_2__. In this way, neurons *σ*_*c*_2__ and *σ*_*c*_3__ receive one spike from each other in each step before *σ*_in_ receives the third spike from the environment. Neuron *σ*_2_ has *y* spikes at the end. When neuron *σ*_in_ receives the third spike, each of the neurons *σ*_*c*_1__, *σ*_*c*_3__, and *σ*_*c*_4__ gets one spike, while *σ*_*c*_2__ receives two spikes. As a result, neuron *σ*_*c*_3__ has an odd number of spikes and the rule cannot be applied. At present, neuron *σ*_*c*_4__ has three spikes, and the rule *a*^3^⟶*a* in neuron *σ*_*c*_4__ fires, which generates one spike and sends it to *σ*_*l*_0__. In this way, it can simulate the instruction *l*_0_ in the next step.

As with the proof of Theorems 1 and 2, the system uses the following numbers of neurons:

  9 neurons for 9 registers
  25 neurons for 25 labels
  5 neurons for the module INPUT
  1 neuron in each SUB instructions and 14 in total
  2 neurons for the module OUTPUT

Therefore, totally 55 neurons are used.

The numbers of neurons can be decreased by exploring some relationships between some instructions of register machine *M*_*u*_′. The following modules are given to reduce the number of neurons in the computation process.

**Figure 10:**
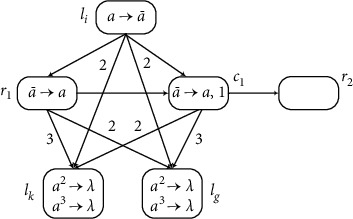
Module SUB-ADD-1: the sequence of the ADD and SUB instructions *l*_*j*_ : (ADD(*r*_2_), *l*_*g*_) and *l*_*i*_ : (SUB(*r*_1_), *l*_*j*_, *l*_*k*_).

**Figure 11:**
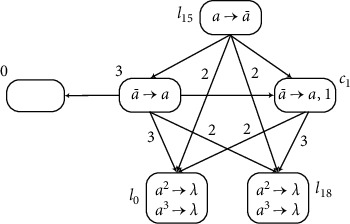
Module SUB-ADD-2: the sequence of the ADD and SUB instructions *l*_20_ : (ADD(0), *l*_0_) and *l*_15_ : (SUB(3), *l*_18_, *l*_20_).

The SUB-ADD instructions can be divided into two cases, depending on the number of spikes placed in register *r*_1_ (the register involved in the SUB instruction). Modules SUB-ADD-1 and SUB-ADD-2 shown in Figures 10 and 11 can simulate the SUB and ADD instructions sequentially. The working process of module SUB-ADD-1 is similar to that of module SUB. When the rule in neuron *σ*_*l*_*i*__ is used and *σ*_*r*_1__ contains at least one spike, neuron *σ*_*r*_1__ cannot fire. Neuron *σ*_*c*_1__ fires by receiving one $\bar{a}$ and then sends one spike to *σ*_*r*_2__. At the end of the computation, neuron *σ*_*l*_*g*__ has one spike, neuron *σ*_*r*_2__ has one spike, and neuron *σ*_*l*_*k*__ is empty. When *σ*_*r*_1__ is empty, neurons *σ*_*r*_2__ and *σ*_*l*_*g*__ are also empty and neuron *σ*_*l*_*k*__ contains one spike. Thus, each pair of SUB-ADD-1 instructions *l*_*i*_ : (SUB(*r*_1_), *l*_*j*_, *l*_*k*_) and *l*_*j*_ : (ADD(*r*_2_), *l*_*g*_) can share a common neuron when *r*_1_ ≠ *r*_2_, and there are totally 6 pairs in *M*_*u*_′:

(2) $$\begin{array}{c} l_{0}:\left(\text{SUB}\left(1\right),l_{1,}l_{2}\right),\\ l_{1}:\left(ADD\left(7\right),l_{0}\right),\\ l_{4}:\left(\text{SUB}\left(6\right),l_{5,}l_{3}\right),\\ l_{5}:\left(ADD\left(5\right),l_{6}\right),\\ l_{6}:\left(\text{SUB}\left(7\right),l_{7,}l_{8}\right),\\ l_{7}:\left(ADD\left(6\right),l_{4}\right),\\ l_{8}:\left(\text{SUB}\left(6\right),l_{9,}l_{0}\right),\\ l_{9}:\left(ADD\left(6\right),l_{10}\right),\\ l_{14}:\left(\text{SUB}\left(5\right),l_{16,}l_{17}\right),\\ l_{16}:\left(ADD\left(4\right),l_{11}\right),\\ l_{22}:\left(\text{SUB}\left(0\right),l_{23,}l_{h}^{\prime }\right),\\ l_{23}:\left(ADD\left(0\right),l_{0}\right). \end{array}$$

By using this module, 6 neurons can be saved. In the same way, the module shown in Figure 10 can simulate the two instructions *l*_15_ and *l*_20_. Neuron *σ*_*l*_20__ can be saved.

**Figure 12:**
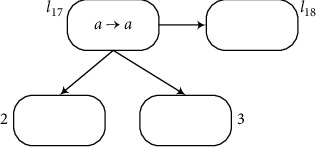
Module ADD-ADD: the sequence of ADD and ADD instructions *l*_17_ : (ADD(2), *l*_21_) and *l*_21_ : (ADD(3), *l*_18_).

The module ADD-ADD shown in Figure 12 can simulate instructions *l*_17_ and *l*_21_. In this way, one neuron can be saved.

**Figure 13:**
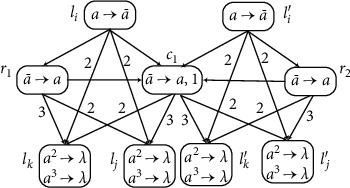
Module SUB-SUB with *r*_1_ ≠ *r*_2_.

The SUB instructions share a common neuron when the labels of their registers are different, as shown in Figure 13. Assume that the simulation of the SUB instruction *l*_*i*_ : (SUB(*r*_1_), *l*_*j*_, *l*_*k*_) starts at time *t*. When neuron *σ*_*l*_*i*__ gets a spike, the rule $a⟶\bar{a}$ fires and sends one anti-spike to *σ*_*r*_1__ and two anti-spikes to *σ*_*l*_*j*__ and *σ*_*l*_*k*__, respectively, at time *t* + 1. Neuron *σ*_*c*_1__ receives an anti-spike at time *t* + 2. Neurons *σ*_*r*_1__, *σ*_*l*_*j*__, *σ*_*l*_*k*__, and *σ*_*c*_1__ work in the same way as those in module SUB shown in Figure 4. Neuron *σ*_*c*_1__ will send three spikes to *σ*_*l*_*k*_′_ and two spikes to *σ*_*l*_*j*_′_, where forgetting rules will be applied. Thus, the instruction *l*_*i*_ : (SUB(*r*_1_), *l*_*j*_, *l*_*k*_) is correctly simulated by this module. The process when starting with instruction *l*_*i*_′ is similar to that described above.

**Figure 14:**
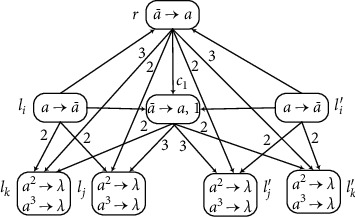
Module SUB-SUB with *r*_1_=*r*_2_.

Two SUB modules dealing with the same register, as shown in Figure 14, can also be proved to work correctly in a similar way. Assume that the instruction *l*_*i*_ : (SUB(*r*_1_), *l*_*j*_, *l*_*k*_) is simulated and one spike is contained in neuron *σ*_*l*_*i*_′_. The process is divided into two cases according to the number of spikes in neuron *σ*_*r*_. When *σ*_*r*_ has at least one spike, the working process of the system is similar to that of module SUB. When *σ*_*r*_ is empty, the rule in neuron *σ*_*c*_1__ cannot be used. Neurons *σ*_*l*_*j*__, *σ*_*l*_*k*_′_, and *σ*_*l*_*j*_′_ are all empty but neuron *σ*_*l*_*k*__ contains one spike. All SUB instructions can be simulated correctly by the module. Therefore, all SUB modules can share a common neuron.

From the above description about the numbers of neurons saved, the system uses the following:

  9 neurons for 9 registers
  17 neurons for 17 labels
  5 neurons for the module INPUT
  1 neuron for all the 14 SUB instructions
  2 neurons for the module OUTPUT

A total of 21 neurons can be saved and the number of neurons in this system can be decreased from 55 to 34. The proof is complete.

### 5.2. The Small Universality as Number Generator

A small universal AWSN P system as a number generator is considered. The process of simulating universal number generators is similar to that of simulating general function computing devices, but the difference between them lies in the module INPUT. The system starts with the spike train 10^*g*(*x*)−1^1 from environment and ends with neuron *σ*_1_ receiving*g*(*x*) spikes. This system is then loaded with an arbitrary number *k*, and neuron *σ*_2_ receives *k* spikes. The number *k* is also the output at the same time as the output spike train 10^*g*(*x*)−1^1, with *g*(*x*) in register 1 and *k* in register 2. Since the output module is not required, that is to say, register 8 is not required, the register machine *M*_*u*_ is simulated. If the computation in *M*_*u*_ halts, the computation can also halt.

**Figure 15:**
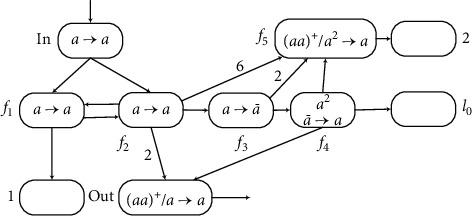
Module INPUT-OUTPUT.

Furthermore, module INPUT and module OUTPUT can be combined. The module INPUT-OUTPUT is shown in Figure 15, and an example is used to prove its feasibility. The label *l*_*h*_′ can also be saved because of module INPUT-OUTPUT. The string 101 is used in module INPUT-OUTPUT, where *g*(*x*)=2 and *k*=4. The computation follows the above working processes of the modules. The results of each step are shown in Table 2.

Assume that *σ*_in_ has one spike at time*t*, and neuron *σ*_*f*_4__ has two spikes. At time*t* + 1, *σ*_*f*_1__ and *σ*_*f*_2__ receive one spike, respectively. From the structure shown in Figure 15, neurons *σ*_*f*_1__ and *σ*_*f*_2__ receive one spike from each other at each step until *σ*_*f*_1__ and *σ*_*f*_2__stop firing. Then *σ*_in_ receives the second spike. Each of neurons *σ*_1_ and *σ*_*f*_3__ receives one spike, *σ*_*f*_5__ receives six spikes, and *σ*_out_ receives two spikes, so that neurons *σ*_*f*_5__ and *σ*_out_ can fire. At time*t* + 3, both *σ*_*f*_1__ and *σ*_*f*_2__ have two spikes, but they cannot fire again. *σ*_*f*_5__ receives six spikes from *σ*_*f*_2__, but *σ*_*f*_5__ also receives two anti-spikes from *σ*_*f*_3__, plus four spikes existing in *σ*_*f*_5__, so that neuron *σ*_*f*_5__ has eight spikes. In addition, neuron *σ*_out_ receives two spikes again, so that there are three spikes contained. Neuron *σ*_*f*_4__ only has one spike because the received anti-spike annihilates one spike. At time*t* + 4, the neuron *σ*_*f*_4__ is empty after receiving an anti-spike. *σ*_*f*_5__ receives two anti-spikes, so that there are four spikes contained in neuron *σ*_*f*_5__, the number of spikes is even, and its rule can fire. At the next step, *σ*_*f*_4__ receives one anti-spike and fires. Neuron *σ*_*f*_5__ consumes two spikes and still can fire. At time*t* + 6, neurons *σ*_*f*_5__ and *σ*_out_ receive one spike from *σ*_*f*_4__, respectively. So, there are 4 spikes in *σ*_out_, meeting the required conditions for firing. Neuron *σ*_*l*_0__ also gets one spike.

The string is read through neuron *σ*_*in*_, and g(*x*) spikes are stored in register 1 when the calculation stops. At the same time , the output number (*t* + 6 − *t* − 2 = 4) is the same as the number stored in register 2. Neuron *σ*_*l*_0__ activates and starts simulating the register machine by simulating modules ADD and SUB. Therefore, through this process, the module INPUT-OUTPUT can be simulated correctly.

Therefore, this system contains the following:

  8 neurons for the 8 registers
  14 neurons for the 14 labels (*l*_*h*_ is saved; 8 neurons are saved by modules SUB-ADD and ADD-ADD)
  1 neuron for 13 SUB instructions
  7 neurons in the module INPUT-OUTPUT

There is a universal AWSN P system having 30 neurons that can be used to perform number generating.

## 6. Conclusions

In this work, a variant of the SN P systems, called the AWSN P systems,is proposed. Because of the use of anti-spikes, the proposed systems are more biologically significant thanSN P systems, with inhibitory spikes in the communication between neurons. An example is used to illustrate the working process of this system. The computational universality is then proved in the case of generating mode and accepting mode, respectively. Finally, the Turing universality of AWSN P systems is proved. The function computing device can be realized by using 34 neurons. Compared with the small universal SN P system using anti-spikes introduced by Song [17], the AWSN P system uses 13 fewer neurons. Compared with the SN P systems with weighted synapses introduced by Pan [34], the AWSN P system uses 4 fewer neurons. The small universality of the ASN P system as number generator is investigated with 30 neurons. Compared with Pan's work [34], the proposed system uses 6 fewer neurons.

The computational universality is proved for AWSN P systems with standard rules. There are three types of spiking rules, $a⟶\bar{a}$, *a*⟶*a*, and $\bar{a}⟶a$, used that are time dependent, and there is one type of forgetting rules, *a*^*c*^⟶*λ*. There are several future research directions. One direction is to investigate whether the computational power will remain the same if only one or two types of spiking rules are used or if the forgetting rules are not used and to investigate whether AWSN P systems can perform better or the same if the spiking rules are not time-dependent. These open problems certainly need further studies. Another future research direction is the application of the proposed systems. There have been studies, such as using SN P systems with learning function for letter recognitions [36]. If the learning function was introduced in AWSN P systems, it may perform better in letter recognitions. Because the use of anti-spikes improves the ability of AWSN P systems to represent and process information, it may solve more practical problems, which still require further research.

## Figures and Tables

**Table 1:** The results of the example.

Step	*σ*_1_	*σ*_2_	*σ*_3_	*σ*_4_
*t*	2	2	0	0
*t* + 1	1	−1	−3	0
*t* + 2	2	0	−3	2
*t* + 3	1	−3	−6	2
*t* + 4	1	−3	0	3 (fires)

**Table 2:** The computation process of the module INPUT-OUTPUT.

Step	*σ*_in_	*σ*_*f*_1__	*σ*_*f*_2__	*σ*_*f*_3__	*σ*_*f*_4__	*σ*_*f*_5__	*σ*_1_	*σ*_2_	*σ*_out_	*σ*_*l*_0__
*t*	1	0	0	0	2	0	0	0	0	0
*t*+1	0	1	1	0	2	0	0	0	0	0
*t*+2	1	1	1	1	2	6	1	0	2 (fire)	0
*t*+3	0	2	2	1	1	8	2	1	3	0
*t*+4	0	2	2	0	0	4	2	2	3	0
*t*+5	0	2	2	0	−1	2	2	3	3	0
*t*+6	0	2	2	0	0	1	2	4	4 (fire)	1

## Data Availability

No datasets were used in this article.
